# Glioblastoma Treatment with Temozolomide and Bevacizumab and Overall Survival in a Rural Tertiary Healthcare Practice

**DOI:** 10.1155/2018/6204676

**Published:** 2018-12-31

**Authors:** Tonia C. Carter, Rafael Medina-Flores, Benjamin E. Lawler

**Affiliations:** ^1^Center for Human Genetics, Marshfield Clinic Research Institute, 1000 North Oak Avenue, Marshfield, WI 54449, USA; ^2^Department of Pathology (Neuropathology), Marshfield Clinic, 1000 North Oak Avenue, Marshfield, WI 54449, USA; ^3^Department of Neurology (Neuro-oncology), Marshfield Clinic, 1000 North Oak Avenue, Marshfield, WI 54449, USA

## Abstract

**Background:**

The efficacy of temozolomide (TMZ) chemotherapy for treating newly diagnosed glioblastoma (GBM), a primary brain tumor with short survival, was demonstrated in a clinical trial in 2005, and since then, the standard-of-care for newly diagnosed GBM has been maximal safe surgery followed by 60 Gray of radiation with concomitant and adjuvant TMZ (standard radiotherapy and TMZ). In 2009, clinical trials also reported on the efficacy of bevacizumab for treating recurrent GBM. We performed a retrospective cohort study to evaluate the impact of treatment regimens on overall survival for patients with GBM at a rural tertiary healthcare practice.

**Methods:**

We retrospectively reviewed the medical records of 307 consecutive, newly diagnosed GBM patients at one institution between 1995 and 2012 and assessed treatment patterns. We also compared overall survival according to the treatment received.

**Results:**

Only 0.6% (1/163) of patients diagnosed before 2005 received standard radiotherapy and TMZ versus 36.1% (52/144) of patients diagnosed since 2005 (*P* < 0.0001). For patients who received standard radiotherapy and TMZ, the median overall survival was 17.0 months versus 7.0 months for patients who received 60 Gray of radiation but no chemotherapy (*P* = 0.0000078). The median overall survival was 15.4 months in the 19 patients treated with bevacizumab monotherapy at first GBM recurrence versus 6.8 months in the 32 patients with no treatment at first GBM recurrence (*P* = 0.00015), but patients who received bevacizumab were younger and more likely to have had a surgical resection and 60 Gray of radiation at diagnosis.

**Conclusions:**

TMZ and bevacizumab therapies were rapidly adopted in a rural tertiary healthcare setting, and patients who received these treatments had increased overall survival. However, advantageous prognostic factors in patients who received bevacizumab at recurrence may have influenced the extent of the increase in overall survival attributed to this treatment.

## 1. Introduction

Glioblastoma (GBM) is an aggressive, infiltrative, primary brain malignancy with a poor prognosis [1]. Median survival without treatment is 2-3 months [2, 3]. Surgical resection to reduce tumor volume and postoperative radiotherapy administered to a total dose of 60 Gray (Gy) in 30 fractions are associated with improved survival [4, 5], and surgical resection with subsequent radiotherapy was used to treat new GBM cases diagnosed before 2005 [6]. In 2005, a prospective, randomized trial showed that adding concurrent and adjuvant temozolomide (TMZ), an alkylating agent that causes DNA damage leading to tumor cell death, to standard postoperative radiotherapy (60 Gy/30 fractions) increased median overall survival from 12.1 to 14.6 months [7]. Therefore, since 2005, maximal safe surgery (biopsy or resection) that preserves performance status, 60 Gy of radiation, and concomitant and adjuvant TMZ chemotherapy is considered the standard treatment for newly diagnosed GBM [7]. However, despite initial treatment, GBM often recurs [8]. Bevacizumab, an angiogenesis inhibitor that can retard tumor growth [9], received provisional approval from the United States Food and Drug Administration (FDA) in 2009 for the treatment of recurrent GBM on the basis of results from two clinical trials that showed progression-free survival increased after bevacizumab treatment of recurrent GBM [10, 11], and received full approval in 2017 [12].

We reviewed the treatment and survival of adult patients consecutively diagnosed with GBM at one rural tertiary healthcare practice between 1995 and 2012, a time period that includes several years before and after TMZ was introduced for newly diagnosed GBM and bevacizumab was approved for the treatment of recurrent GBM. To determine whether these treatments were translated into clinical practice in a rural healthcare setting, we compared treatment type before and after TMZ was introduced and examined the details of GBM treatment in patients who received bevacizumab. To assess whether the treatments showed evidence of a survival benefit, we compared overall survival according to use of the standard-of-care regimen at diagnosis or use of bevacizumab at first GBM recurrence and also evaluated the treatments for independent associations with overall survival. Because many GBM patients are elderly [13], we also compared treatment type and survival according to patient age, as clinical trials of treatments for GBM have often excluded older patients [5, 7].

## 2. Materials and Methods

### 2.1. Subjects

Patients were identified retrospectively from medical records at Marshfield Clinic, a multispecialty clinic with affiliated hospitals in Wisconsin, USA, that serves a predominantly rural population. Patients were included in the study if they were newly diagnosed with GBM between 1995 and 2012 and at least 18 years of age at the time of diagnosis. Pathology reports and available histopathological material were reviewed by a neuropathologist to confirm the diagnosis of GBM (World Health Organization grade IV astrocytoma) for each patient. Patients without histological confirmation of GBM or who were diagnosed with GBM at autopsy only were excluded. The research was carried out according to the principles outlined in the Declaration of Helsinki (1964) and all subsequent revisions, and the Institutional Review Board of the Marshfield Clinic Research Institute approved the study.

### 2.2. Healthcare Setting

The primary service area of Marshfield Clinic comprises 28 counties in central and northern Wisconsin and Gogebic county in Michigan. In 20 of the 29 counties, ≥ 60% of the population lives in a rural area (city or town with < 2,500 people), according to the 2010 United States Census [14, 15]. For the year 2012, the median (interquartile range) distance between patient residential address in the medical record and the Marshfield Clinic facility where healthcare was received was 19.3 (0.0-43.5) kilometers. The distance to a Marshfield Clinic facility was calculated using an algorithm that matched patient and facility addresses to zip code regions, assigned each address to the center point of its matching zip code region, and calculated the distance between zip code center points. Five linear accelerators, each located at one of five different Marshfield Clinic facilities within the Marshfield Clinic primary service area, were available for radiotherapy services. The team of physicians providing healthcare to GBM patients included specialists in neuro-oncology, neuro-pathology, neuro-surgery, radiation-oncology, neuro-radiology, and neuro-psychology.

### 2.3. Demographic and Clinical Data

Medical records were reviewed to obtain data on patient age at GBM diagnosis, sex, race, year of GBM diagnosis, presenting symptoms, comorbidities, extent of surgery, tumor location and size, use of radiotherapy and chemotherapy, tumor recurrence, and date of death. Comorbidities were represented in this study by the Charlson comorbidity score, a weighted sum of comorbid conditions based on the risk of mortality for each condition [16]. A score of zero indicates no comorbidities, and the higher the score, the greater the burden of comorbidities. The extent of surgery was based on surgery reports, and tumor size and location were obtained from neuroimaging reports. Tumor size was defined as the largest diameter of contrast-enhancing tumor. Overall survival was the only survival outcome analyzed and was measured from the date of surgery for GBM until either the date of death or December 31, 2015.

### 2.4. Statistical Analysis

Data were summarized as mean ± standard deviation for parametric variables and median (interquartile range) for nonparametric variables. Groups were compared using the chi-squared test for categorical data and the Wilcoxon rank sum test for continuous data.

No patients were lost to follow-up and data were censored for patients alive on December 31, 2015. For bivariate analyses, survival curves were generated using the Kaplan-Meier method and were compared using the log-rank test. Cox proportional hazards regression analysis was used to determine whether TMZ and bevacizumab therapies were independently associated with survival in a multiple regression model. All variables associated with survival in bivariate analyses (*P* < 0.10) were included as covariates in the regression model. Statistical significance was considered as a two-sided* P* value < 0.05.

## 3. Results

### 3.1. Patient and Clinical Characteristics

Between 1995 and 2012, 307 adult patients were newly diagnosed with GBM. The mean age at diagnosis was 64.9 ± 13.9 years; 59% of patients were male, 96% were Caucasian, and approximately 50% had comorbid conditions (Table 1). Fifty-three percent of patients were diagnosed before 2005, the year when TMZ chemotherapy for newly diagnosed GBM was introduced. One or more presenting symptoms were reported for every patient, and headaches were the most commonly reported symptom. The tumor was surgically resected in 58.6% of patients and 36.8% had a biopsy only. The reason for not receiving a surgical resection was noted in the medical records of 22.1% (25/113) of patients who received a biopsy. Of the 25 patients, 12 had a tumor that was deemed not able to be resected because the tumor was located in or near eloquent brain areas or was bilateral, another 10 had severe neurological deficits and it was considered unlikely that a surgical resection would reverse the deteriorating clinical course of these patients, and the remaining three patients declined any type of treatment and chose to receive supportive care instead. Radiotherapy was documented in the medical records of 218 patients and 67.4% (*n* = 147) of these patients received 60 Gy of radiation. The extent of surgery and the radiation dose were unknown for some patients who received these treatments at other institutions. Of the 176 patients who had chemotherapy, 130 (73.9%) were treated with TMZ. Tumors were most often located in the frontal, temporal, and parietal areas of the brain and often occupied more than one lobe. GBM was less common in the thalamus, cerebellum, and brainstem. Tumor size was available for 254 patients, and mean tumor size was 4.4 ± 1.6 cm.

### 3.2. Treatment before and after Widespread Use of Temozolomide

We defined standard treatment as maximal safe surgery followed by standard radiotherapy (60 Gy), completion of concomitant TMZ, and completion of at least one cycle of adjuvant TMZ. The percentage of patients who received any radiotherapy, standard radiotherapy, any chemotherapy, TMZ chemotherapy, or standard treatment was higher when the diagnosis occurred during 2005-2012 than during 1995-2004 (Table 2). Of the 112 patients who received TMZ after a GBM diagnosis in 2005-2012, 103 (92.0%) started concomitant TMZ and 69 (61.6%) started adjuvant TMZ. Sixty (53.6%) of the 112 patients completed concomitant TMZ and at least one cycle of adjuvant TMZ, 27 (24.1%) patients completed concomitant TMZ but did not receive any adjuvant TMZ, nine (8.0%) patients completed at least one cycle of adjuvant TMZ without having received any concomitant TMZ, and 16 (14.3%) patients started but did not complete concomitant TMZ and also did not receive any adjuvant TMZ. The reasons for not completing concomitant or adjuvant TMZ included disease progression (*n* = 27), toxic effects (*n* = 13), patient refusal to continue treatment (*n* = 5), and the poor medical condition of the patient (*n* = 2). The reasons were unknown for another five patients. Thirty-two (22.2%) of the 144 patients diagnosed during 2005-2012 were not treated with TMZ. The reasons for not receiving TMZ included the decision of the patient to decline further treatment and to continue with supportive care only (*n* = 19 patients), the development of severe illness after surgery that led to a worsening of the medical condition of the patient (*n* = 2 patients), and the presence of complications due to serious comorbidites (*n* = 1 patient). The reason for the remaining 10 of the 32 patients was unknown because a reason was not documented in their medical records.

### 3.3. Treatment by Age at Diagnosis

A comparison between patients ≥ 65 years at diagnosis and those < 65 years at diagnosis showed that older patients experienced more comorbid conditions, were less likely to have surgical resection, 60 Gy of radiation, TMZ chemotherapy, or standard treatment, and were more likely to have no radiotherapy and no chemotherapy than younger patients (Table 3).

### 3.4. Bevacizumab Treatment

Sixty patients received bevacizumab treatment and for 10 (16.7%) of these patients, bevacizumab was administered before GBM recurrence. One of the 10 patients was included in a clinical trial that used bevacizumab as first-line treatment. For the other nine patients, subtle increases in tumor size were observed following the start of radiotherapy and chemotherapy, and patients were given bevacizumab because it was uncertain whether these changes were due to tumor progression or the effects of treatment. The other 50 (83.3%) of the 60 patients were treated with bevacizumab after GBM recurrence, determined from brain imaging reports that indicated an increase in tumor size or the appearance of new lesions. Forty-three (86.0%) of the 50 patients received bevacizumab at the first recurrence and seven (14.0%) at the second recurrence. Patients who were treated with bevacizumab also received radiotherapy and TMZ during the course of treatment (Table 4). Bevacizumab was received at the first or second GBM recurrence by 24.1% (33/137) of patients diagnosed at age < 65 years compared with 10.0% (17/170) of patients diagnosed at age ≥ 65 years (*P* = 0.00089).

### 3.5. Patient Overall Survival

Three hundred and one (98.0%) of the 307 patients were deceased at last follow-up and the median survival in our patient population was 7.6 (3.2-14.9) months. The percentage of patients that survived one, two, and five years was 32.6%, 11.4%, and 2.3%, respectively. Median survival in the 53 patients who received the standard treatment was 17.0 (13.3-27.1) months compared with 7.0 (4.4-11.5) months in the 29 patients who received 60 Gy of radiation but no chemotherapy (*P* = 0.0000078). The median survival in patients ≥ 65 years was 5.2 (2.0-9.5) months compared with 12.0 (5.9-18.6) months in patients < 65 years (*P* < 0.0001). When analyzed by type of treatment received, the median overall survival did not differ by age group for patients who received the standard treatment and for those who received no treatment (biopsy only) (Table 5). However, the median overall survival was significantly extended in younger patients who received TMZ but did not complete the standard regimen and those who received treatment that did not include TMZ.

### 3.6. Overall Survival with Bevacizumab Treatment

**Figure 1:**
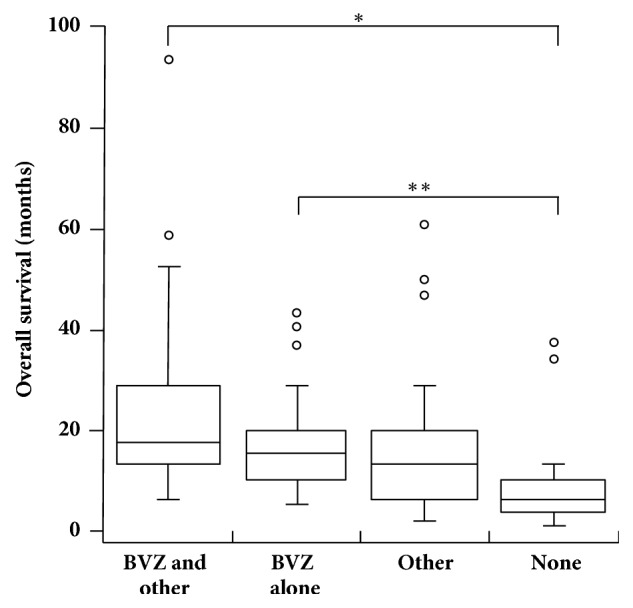
Overall survival according to bevacizumab treatment at first glioblastoma recurrence. Sample sizes were* n* = 24 for patients who received bevacizumab and other agents (BVZ and other),* n* = 19 for patients who received bevacizumab alone (BVZ alone),* n* = 22 for patients who received only non-bevacizumab treatment (Other), and* n* = 32 for patients who received no treatment (None). The distance spanned by the bottom and top of each box represents the interquartile range, the enclosed line represents the 50^th^ percentile, and the whiskers stretch to the data point that is not > 1.5 times the interquartile range from the box. Data points that fell outside the range of the whiskers were represented by small, open circles. ^*∗*^*P* = 0.0000033 and ^*∗∗*^*P* = 0.00015 for paired comparisons between treatment groups using Wilcoxon rank sum test. Abbreviation: BVZ, bevacizumab.

Because the 43 patients treated with bevacizumab at first GBM recurrence had all received some radiotherapy and TMZ chemotherapy at diagnosis, we compared survival in these patients with survival in the 54 patients who received some radiotherapy and TMZ chemotherapy at diagnosis but no bevacizumab at first GBM recurrence. The groups included in the analysis were the 24 patients who received bevacizumab with other chemotherapy and/or radiotherapy at first GBM recurrence, the 19 patients who received bevacizumab alone at first GBM recurrence, 22 patients who received other (non-bevacizumab) treatment at first GBM recurrence, and 32 patients who received no treatment at first GBM recurrence. The median survival was 17.8 (13.5-29.2) months in patients treated with bevacizumab and other agents, 15.4 (9.2-19.7) months in patients treated with bevacizumab alone, 13.6 (6.1-20.0) months in patients who received other (non-bevacizumab) treatment only, and 5.6 (3.4-9.8) months in patients who received no treatment at first GBM recurrence (Figure 1). Patients who received no treatment had a median survival that was significantly shorter than that of patients treated with bevacizumab and other agents (*P* = 0.0000033) and patients treated with bevacizumab alone (*P* = 0.00015) (Figure 1). The median survival in patients who received other (non-bevacizumab) treatment was not significantly different from that of patients who received bevacizumab and other agents (*P* = 0.064) or patients who received bevacizumab alone (*P* = 0.48). Also, the median survival in patients treated with bevacizumab and other agents did not differ significantly from that of patients treated with bevacizumab alone (*P* = 0.17).

We considered the possibility that factors other than bevacizumab treatment, such as age, extent of surgery, and dose of radiation at diagnosis, may have contributed to the difference in median overall survival between patients who received bevacizumab and those who received no treatment at first GBM recurrence. The median age at diagnosis was 61.9 (54.7-67.4) years in the 43 patients who received bevacizumab treatment (with or without other agents) at first recurrence versus 70.6 (63.8-79.9) years in the 32 patients with no treatment at first recurrence (*P* = 0.0011). The percentage of patients who had a surgical resection at diagnosis was 67.4% (29/43) for patients treated with bevacizumab at first recurrence versus 40.6% (13/32) for patients with no treatment at first recurrence (*P* = 0.021). Additionally, the percentage of patients who were given 60 Gy of radiation at diagnosis was 83.7% (36/43) for patients with bevacizumab treatment at first recurrence compared with 50.0% (16/32) for patients with no treatment at first recurrence (*P* = 0.0017). Because of the small sample size, analyses were not performed for the group of seven patients treated with bevacizumab after the second GBM recurrence.

### 3.7. Factors Associated with Survival

In bivariate analyses, decreased survival was associated with age ≥ 65 years at diagnosis, no surgical resection of the tumor, < 60 Gy of radiation or no radiotherapy, non-TMZ chemotherapy or no chemotherapy, a Charlson comorbidity score > 0, and tumor location in the occipital region or corpus callosum (Table 6). Patient sex, type of presenting symptoms, tumor size > 5 cm, and tumor location in regions other than the occipital region or corpus callosum were not associated with survival (*P* > 0.10). In Cox proportional hazards regression analysis, TMZ chemotherapy with or without bevacizumab was independently associated with increased survival (Table 6).

## 4. Discussion

At our institution, the standard treatment became more widely used soon after demonstration of the ability of TMZ chemotherapy to increase survival in a clinical trial, and survival was longer in patients who received the standard treatment, consistent with reports from other population-based studies [2, 17, 18]. Patients who were ≥ 65 years at diagnosis were less likely to receive the standard treatment, and for both older and younger patients, survival with the standard treatment was longer than survival with other treatment or no treatment, indicating that improved survival after standard treatment was not restricted to younger patients. The lower probability of receiving surgical resection and adjuvant treatment with increasing patient age [2, 18, 19] as well as improved survival for older patients who received the standard treatment [20, 21] have been documented previously. We also observed that TMZ chemotherapy (with or without use of bevacizumab) was an independent predictor of survival in our study population and that tumor location in the occipital region or corpus callosum was associated with shorter survival in bivariate analyses. Surgical resection is often not performed for tumors in the occipital region or corpus callosum because aggressive surgery can lead to loss of function, and in the regression model adjusted for extent of surgery and other covariates, tumor location in these brain regions was no longer associated with survival.

Of the 307 patients in our study, 36.8% received a biopsy, and of the 144 patients diagnosed during the period 2005-2012, 77.8% received radiotherapy, 77.8% received some TMZ, and 41.7% received concomitant and adjuvant TMZ. Similar findings have been reported in previous studies of patterns of care for GBM but estimates varied among studies. In these studies, 4.6-44.4% of patients received a biopsy [18, 22–25], and since 2005, 72.2-80.0% of patients received radiotherapy [18, 25, 26], 59.9-70.7% of patients received some TMZ chemotherapy [17, 18, 26, 27], and 51.2-57.0% of patients received concomitant and adjuvant TMZ [18, 27]. Also, of the 144 patients diagnosed during 2005-2012 at our institution, 18.1% received neither radiotherapy nor chemotherapy, which is comparable to the range of 20.0-22.3% reported in previous studies for patients diagnosed since 2005 and who received no radiotherapy and no chemotherapy [18, 25].

Our findings that a substantial fraction of the study population received a biopsy as maximal safe surgery and as much as 22.2% of patients diagnosed in 2005-2012 were not given TMZ motivate consideration of the factors that influence decisions about GBM treatment at our institution. The available medical record data indicated that the presence of a tumor not amenable to resection, the poor medical condition of the patient, and patient preference were among the factors involved. In a study of GBM patients diagnosed after 2005 in Spain, reasons why patients received no radiotherapy and no chemotherapy after surgery included low performance status, surgical complications, tumor-related symptoms, probable tumor progression, and patient decision [18]. Information on why patients in our study did not get treatment was limited because the reasons were often not stated in the medical records. However, documenting and evaluating these reasons potentially could facilitate identification of the main factors that influence physicians and patients when making treatment decisions about GBM, enhance understanding of how patients make treatment decisions during a crisis such as a GBM diagnosis, and reveal opportunities for improving the oncological care of GBM patients at our institution.

Bevacizumab at first GBM recurrence was associated with a statistically significant increase in overall survival when compared with no treatment in this study. In a systematic review of 17 other studies, a gain in overall survival with use of bevacizumab compared with use of other treatment for recurrent GBM has been observed [28]. Median overall survival was 39.5 ± 6.2 weeks for bevacizumab combination therapy and 36.2 ± 3.8 weeks for bevacizumab monotherapy compared with 22.4± 4.3 weeks for other treatment for recurrent GBM (based on five studies of other treatment). However, many of these studies did not have adequate control groups of patients unexposed to bevacizumab and, in nine of the studies, only patients with good performance status were included [28]. In our study population, several factors that were independent predictors of longer overall survival in GBM (younger age, surgical resection, and standard postoperative radiotherapy at diagnosis) were more common in patients who received bevacizumab than in patients who received no treatment at first GBM recurrence. This indicates a need for caution when interpreting our study's findings because our comparison between bevacizumab treatment and no treatment probably did not provide an unbiased estimate of the effect of bevacizumab treatment at GBM recurrence on overall survival. In two randomized, controlled trials of bevacizumab treatment at GBM recurrence, overall survival did not differ significantly between patients on bevacizumab monotherapy and those on lomustine monotherapy, and whereas progression-free survival increased for patients who received the combination therapy of bevacizumab added to lomustine compared with those who received lomustine monotherapy, the combination therapy did not result in a gain in overall survival [29, 30]. Therefore, the efficacy of bevacizumab for the treatment of recurrent GBM is in question.

The limited options currently available for the treatment of recurrent GBM also include tumor-treating fields, electric fields that are delivered by a noninvasive, portable device and that can physically disrupt cell division leading to antimitotic effects on tumor cells [31]. The FDA approved this therapy for recurrent glioblastoma in 2011 based on the results of a randomized, controlled trial which showed that median overall survival was comparable between tumor-treating fields and chemotherapy regimens and that tumor-treating fields provided the added benefit of reduced toxicity and improved quality of life [32]. Following this, the FDA also approved tumor-treating fields therapy for newly diagnosed GBM based on observations in a randomized, controlled trial that overall survival and progression-free survival were longer when patients received tumor-treating fields with maintenance chemotherapy compared with maintenance chemotherapy only at GBM diagnosis [33]. Although tumor-treating fields therapy was demonstrated to be efficacious in this trial, median overall survival in newly diagnosed patients who received the therapy was no longer than two years [33]; therefore, the development of new treatments that further improve patient outcomes remains a priority for GBM research.

An advantage of this study was the availability of clinical data for an unselected patient population seen in routine clinical practice in a rural tertiary healthcare center. These patients had less opportunity to participate in research treatment protocols established in many academic centers to treat newly diagnosed or recurrent GBM. Yet, data on this patient population allowed us to assess changes in GBM treatment that occurred once reports demonstrating the efficacy of new treatments became available and to evaluate the effects of the new treatments on survival in patients who were not required to meet any specific criteria such as those used to select patients for enrollment in clinical trials. Disadvantages included the lack of data on several factors known to be associated with prognosis including performance status [34], marital status [4], and molecular markers such as promoter methylation of the O-6 methylguanine-DNA methyltransferase gene [35] and mutations in the isocitrate dehydrogenase 1 gene [36]. Molecular testing was performed only at the discretion of clinicians to aid histological classification of the tumor; however, our institution plans to incorporate a next-generation sequencing gene panel tailored for gliomas [37] into the diagnostic process for GBM in the future. Another disadvantage was the limited data available on tumor progression, residual tumor volume after surgical resection, the decision-making process for selecting treatment, and quality of life. Additionally, because this study was retrospective, direct causal relationships between treatment type and survival should not be inferred.

## 5. Conclusions

This study captured one rural healthcare institution's transition to treating GBM with TMZ and bevacizumab once these therapies were introduced. The standard regimen of maximal safe surgery, standard radiotherapy, and concomitant and adjuvant TMZ chemotherapy was more often used to treat patients diagnosed since 2005 than patients diagnosed in previous years, and patients who received this regimen survived longer than patients who received standard radiotherapy without TMZ chemotherapy. Overall survival improved with bevacizumab treatment compared with no treatment at first GBM recurrence but because patients who received bevacizumab at first recurrence had more favorable prognostic factors than patients who received no treatment at first recurrence, the magnitude of the observed increase in survival with bevacizumab treatment may be biased by these prognostic factors. Few studies of GBM in rural populations have been done and ours supports continued use of standard radiotherapy with concomitant and adjuvant TMZ chemotherapy for treatment of GBM in a rural tertiary healthcare setting.

## Figures and Tables

**Table 1:** Characteristics of patients diagnosed with glioblastoma.

Characteristic	*n* (%)
Age at diagnosis (years)	
18-39	15 (4.9)
40-49	29 (9.4)
50-59	58 (18.9)
60-69	85 (27.7)
70-79	75 (24.4)
≥ 80	45 (14.7)
Year of diagnosis	
1995-1999	89 (29.0)
2000-2004	74 (24.1)
2005-2009	89 (29.0)
2010-2012	55 (17.9)
Male	181 (59.0)
Race	
White	295 (96.0)
African-American	1 (0.3)
Asian	2 (0.7)
American Indian or Alaska native	2 (0.7)
Unknown	7 (2.3)
Charlson comorbidity score	
0	155 (50.5)
1	29 (9.5)
2	60 (19.5)
≥ 3	63 (20.5)
Presenting symptoms	
Headaches	160 (52.1)
Seizures	75 (24.4)
Nausea/vomiting	48 (15.6)
Sensory deficit^1^	56 (18.2)
Motor deficit^2^	137 (44.6)
Confusion/memory loss	175 (57.0)
Extent of surgery	
Resection	180 (58.6)
Biopsy	113 (36.8)
Unknown	14 (4.6)
Radiation dose	
60 Gy	147 (47.9)
< 60 Gy	47 (15.3)
Unknown dose	24 (7.8)
No radiotherapy	89 (29.0)
Chemotherapy	
Temozolomide only	44 (14.3)
Temozolomide and other agents	86 (28.0)
Non-temozolomide agents only	46 (15.0)
No chemotherapy	131 (42.7)
Tumor location - supratentorial	
Frontal	124 (40.4)
Parietal	105 (34.2)
Temporal	136 (44.3)
Occipital	43 (14.0)
Corpus callosum	42 (13.7)
Thalamus	13 (4.2)
Tumor location - infratentorial	
Cerebellum	11 (3.6)
Brainstem	6 (2.0)
Tumor location - unknown	4 (1.3)

^1^A sensory deficit was defined as decreased sensation to any stimulus. ^2^A motor deficit was defined as decreased strength and/or difficulty with movement or coordination.

**Table 2:** Treatment with surgical resection, radiotherapy, and chemotherapy by time period of glioblastoma diagnosis.

Treatment	Time period of diagnosis		*P*-value^1^
	1995-2004	2005-2012	
	*n* = 163	*n* = 144	
	*n* (%)	*n* (%)	
Surgical resection	100 (61.3)	80 (55.6)	0.30
Any radiotherapy	106 (65.0)	112 (77.8)	0.014
60 Gray of radiation	66 (40.5)	81 (56.3)	0.0058
Any chemotherapy	63 (38.7)	113 (78.5)	< 0.0001
Temozolomide chemotherapy	18 (11.0)	112 (77.8)	< 0.0001
Other (non-temozolomide) chemotherapy	45 (27.6)	1 (0.7)	< 0.0001
Concomitant and adjuvant temozolomide chemotherapy	2 (1.2)	60 (41.7)	< 0.0001
Standard treatment^2^	1 (0.6)	52 (36.1)	< 0.0001
Radiotherapy without chemotherapy	50 (30.7)	5 (3.5)	< 0.0001
No radiotherapy and no chemotherapy	50 (30.7)	26 (18.1)	0.011

^1^Chi-squared test used to compare the percentage of patients between the two time periods.

^2^Standard treatment was maximal safe surgery, postoperative administration of 60 Gray of radiation, completion of concomitant temozolomide chemotherapy, and completion of at least one cycle of adjuvant temozolomide.

**Table 3:** Glioblastoma patient comorbidities and treatments according to age at diagnosis.

Characteristic	Age at diagnosis		*P*-value^1^
	< 65 years	≥ 65 years	
	*n* = 137	*n* = 170	
	*n* (%)	*n* (%)	
Charlson comorbidity score			< 0.0001
0	90 (65.7)	65 (38.2)	
1	8 (5.8)	21 (12.4)	
2	23 (16.8)	37 (21.8)	
≥ 3	16 (11.7)	47 (27.6)	
Extent of surgery			0.00065
Resection	89 (65.0)	91 (53.5)	
Biopsy	37 (27.0)	76 (44.7)	
Unknown	11 (8.0)	3 (1.8)	
Radiation dose			0.00026
60 Gy	84 (61.3)	63 (37.1)	
< 60 Gy	18 (13.1)	29 (17.0)	
Unknown dose	9 (6.6)	15 (8.8)	
No radiotherapy	26 (19.0)	63 (37.1)	
Chemotherapy			< 0.0001
Temozolomide with or without other agents	70 (51.1)	60 (35.3)	
Non-temozolomide agents only	30 (21.9)	16 (9.4)	
No chemotherapy	37 (27.0)	94 (55.3)	
Standard treatment	38 (27.7)	15 (8.8)	< 0.0001
No radiation and no chemotherapy	20 (14.6)	56 (32.9)	0.00021

^1^Chi-squared test used to compare characteristics between the two age groups.

**Table 4:** Treatments for glioblastoma administered to 60 patients who received bevacizumab therapy.

Bevacizumab treatment sub-groups (number of subjects)	Period when treatment for glioblastoma was received	Treatment received for glioblastoma				
		Surgical resection	Any radiotherapy	Temozolomide	Bevacizumab	Other chemotherapy
		*n* (%)	*n* (%)	*n* (%)	*n* (%)	*n* (%)
Bevacizumab received before tumor recurrence (*n* = 10)	At diagnosis	9 (90.0)	10 (100.0)	10 (100.0)	10 (100.0)	2 (20.0)
	At first recurrence	2 (20.0)	0 (0.0)	0 (0.0)	2 (20.0)	3 (30.0)

Bevacizumab received at first tumor recurrence (*n* = 43)	At diagnosis	29 (67.4)	43 (100.0)	43 (100.0)	0 (0.0)	0 (0.0)
	At first recurrence	0 (0.0)	2 (4.7)	9 (20.9)	43 (100.0)	19 (44.2)

Bevacizumab received at second tumor recurrence (*n* = 7)	At diagnosis	7 (100.0)	6 (85.7)	5 (71.4)	0 (0.0)	2 (28.6)
	At first recurrence	7 (100.0)	3 (42.9)	6 (85.7)	0 (0.0)	1 (14.3)
	At second recurrence	0 (0.0)	1 (14.3)	0 (0.0)	7 (100.0)	5 (71.4)

**Table 5:** Overall survival according to treatment for glioblastoma and age at diagnosis.

Treatment received	< 65 years of age at diagnosis		≥ 65 years of age at diagnosis		*P*-value^1^
	*n*	Overall survival (months)	*n*	Overall survival (months)	
Standard radiotherapy with concomitant and adjuvant temozolomide	38	18.9 (13.7-29.4)	15	16.4 (10.0-19.1)	0.14
Other treatment including temozolomide	32	12.5 (8.1-19.6)	45	6.5 (3.9-13.0)	0.0036
Other non-temozolomide treatment	55	10.2 (5.4-13.8)	76	5.5 (2.1-8.6)	0.0013
No treatment	12	1.9 (1.0-4.1)	34	1.5 (0.6-3.5)	0.40

^1^Wilcoxon rank sum test used for comparing overall survival between the two age groups.

**Table 6:** Factors associated with survival in patients with glioblastoma.

Factor	Log rank test *P*-value^1^	Hazard ratio	95% Confidence interval	*P*-value
Age at diagnosis (years)^2^	< 0.0001	1.02	1.01, 1.03	0.00015
Extent of surgery (Ref: Biopsy)	< 0.0001			
Resection		0.52	0.40, 0.67	< 0.0001
Unknown		0.63	0.35, 1.13	0.12
Radiation dose (Ref: No radiotherapy)	< 0.0001			
60 Gy		0.67	0.48, 0.93	0.015
< 60 Gy		0.98	0.66, 1.46	0.93
Unknown		0.90	0.54, 1.48	0.67
Chemotherapy (Ref: No chemotherapy)	< 0.0001			
Temozolomide and bevacizumab		0.32	0.22, 0.49	< 0.0001
Temozolomide without bevacizumab		0.58	0.41, 0.82	0.0018
Non-temozolomide agents only		0.68	0.46, 1.01	0.054
Charlson comorbidity score^2^	0.049	1.06	0.99, 1.13	0.12
Tumor located in occipital region – Yes/No (Ref: No)	0.0044	1.33	0.95, 1.86	0.10
Tumor located in corpus callosum – Yes/No (Ref: No)	0.00066	1.27	0.90, 1.79	0.17

^1^Age at diagnosis and Charlson comorbidity score were categorized to perform the log rank test. For age at diagnosis, two categories were compared: < 65 years and ≥ 65 years. For Charlson comorbidity score, three categories were compared: 0, 1-2, and ≥ 3.

^2^Age at diagnosis and Charlson comorbidity score were continuous variables in the Cox proportional hazards regression model.

## Data Availability

The data used to support the findings of this study are available from the corresponding author upon request.
